# The epithelial-mesenchymal transition induced by transcription factor LEF-1 is independent of β-catenin

**DOI:** 10.1016/j.bbrep.2018.06.003

**Published:** 2018-06-12

**Authors:** Wakako Kobayashi, Masayuki Ozawa

**Affiliations:** Department of Biochemistry and Molecular Biology, Graduate School of Medical and Dental Sciences, Kagoshima University, 8-35-1 Sakuragaoka, Kagoshima 890-8544, Japan

**Keywords:** EMT, epithelial–mesenchymal transition, LEF-1, lymphoid-enhancer–binding factor 1, TCF, T-cell factor, KO, knockout, CRISPR, clustered regularly interspaced short palindromic repeats, Cas9, CRISPR-associated 9, PAM, protospacer-adjacent motif, DKO, double knockout, EMT, LEF-1, β-catenin, CRISPR/Cas9, Knockout

## Abstract

Transcription factor lymphoid-enhancer–binding factor 1 (LEF-1) is a key molecule in the Wnt/β-catenin signaling pathway. *Slug* is one of the Wnt/β-catenin target genes and can induce epithelial–mesenchymal transition (EMT). Previously, we have shown that not only wild-type LEF-1 but also LEF-1 lacking the amino-terminal β-catenin–binding region can induce EMT, suggesting that LEF-1 acts independently of β-catenin. Because it has been reported that LEF-1 interacts with β-catenin outside the amino-terminal domain, namely, in the middle part of the molecule, the possible participation of β-catenin has not been formally ruled out. To determine the involvement of β-catenin in the LEF-1–induced EMT, we produced MDCK cells with a deletion of the β-catenin gene and then expressed LEF-1 in the cells. We found that LEF-1 induced EMT in those cells. In the absence of β-catenin, γ-catenin has been shown to take over the role of β-catenin. To examine this possibility, we first established MDCK cells with a double knockout of β-catenin and γ-catenin genes and then expressed LEF-1 in these cells. We found that LEF-1 can induce EMT in these cells; therefore, we conclude that neither β-catenin nor γ-catenin expression is necessary for the LEF-1–mediated induction of EMT.

## Highlights

• LEF-1 is a key molecule in the canonical β-catenin-dependent Wnt signaling pathway.
• LEF-1 can induce EMT.
• LEF-1 can induce EMT in cells with a double knockout of β-catenin and γ-catenin genes.
• Neither β-catenin nor γ-catenin expression is necessary for the induction of EMT.

## 1 Introduction

Epithelial–mesenchymal transition (EMT) is known as one of the essential steps for tissue remodeling, organ development, wound healing, and cancer metastasis [1], [2], [3], [4]. When EMT is induced, epithelial characteristics are lost and mesenchymal properties are acquired. EMT-related transcription factors—Snail, Slug, and ZEB1/2—suppress the expression of epithelial markers such as E-cadherin, while increasing expression of mesenchymal markers N-cadherin and fibronectin and augmenting cell motility and the invasive potential.

Members of the lymphoid-enhancer–binding factor 1/T-cell factor (LEF-1/TCF) family are key transcription factors that interact with β-catenin and activate Wnt/β-catenin signaling [5]. They regulate the cell cycle–related gene cyclin D1, cell growth–related gene *c-myc*, and EMT-related gene *SNAI2 (Slug)* [6], [7]. On the other hand, there are some reports that LEF-1 can act without interacting with β-catenin in lung adenocarcinomas, small B-cell lymphomas, and sebaceous skin tumors [8], [9], [10]. Expression of LEF-1 lacking the amino-terminal β-catenin–binding domain (ΔNLEF-1) leads to sebaceous skin tumors in mice [11]. In fact, we found that the expression of ΔNLEF-1 induces EMT in MDCK cells [12]. These observations have suggested that β-catenin is not necessary for the LEF-1–mediated induction of EMT. Nonetheless, there is a report that in addition to the well-established amino-terminal β-catenin–binding domain of LEF-1, β-catenin binds to another site of LEF-1: residues 150–175 [13]. Because of these findings, the possibility of involvement of β-catenin in EMT induction is a contentious topic.

It is known that γ-catenin (also known as plakoglobin) is a scaffold protein at the adherens junction and in the desmosome [14], [15] and is highly homologous to β-catenin. Disruption of the β-catenin gene in F9 cells (via a gene knockout; KO) does not affect cell–cell adhesion and cell morphology [17]. Additional inactivation of the γ-catenin gene in these cells disrupts cell–cell adhesion and induces morphological alterations [17]. E-cadherin expression is downregulated and cytoplasmic aggregates of E-cadherin are frequently observed [17]. In Wnt signaling cascades, γ-catenin can act as an alternative to β-catenin. Indeed, in β-catenin–deficient cells, γ-catenin, instead of β-catenin, plays a key role in the transcriptional activity of *TCF* and *LEF-1* [16].

To determine the involvement of β-catenin in LEF-1–induced EMT, we established MDCK cells with disruption of the β-catenin gene using the CRISPR/Cas9 gene editing system and then introduced a LEF-1 expression vector. We found that expression of LEF-1 induces EMT in these cells. We also generated MDCK cells in which β-catenin and γ-catenin genes were disrupted to rule out the possible compensation of β-catenin by γ-catenin. We found that LEF-1 induces EMT in these cells. These results revealed that LEF-1 induces EMT independently of β-catenin and γ-catenin.

## 2 Materials and methods

### 2.1 Plasmids and guide RNA (gRNA) synthesis

Mouse LEF-1 cDNA was kindly provided by Rolf Kemler (Max Planck Institute for Immunobiology, Germany). HA-tagged LEF-1 expression vector (pCAGGS-LEFHA) has been described previously [12]. A CRISPR/Cas9 knockout vector (pCGSapI) was kindly provided by Takayuki Sakurai (University of Sinshu, Japan). The pCGSapI vector includes human Cas9 cDNA under control of the CAG promoter and expresses gRNA via the U6 promoter [18]. The pCGSapI vector was digested with the SapI enzyme. To construct the KO vectors for β-catenin and γ-catenin, we selected a 20-base sequence upstream of the PAM site (NGG) for β-catenin in exon 4 of the canine *CTNNB1* gene, and for γ-catenin, in exon 3 of the canine *JUP* gene. The sequences of oligonucleotides were as follows: β-catenin gRNA, 5′-ACCGGAAACAGCTCGCTGTACTGCG-3′ and 5′-AAACGCAGTACAGCGAGCTGTTTCC-3′; γ-catenin gRNA, 5′-ACCGCACCAAACTGCTCAACGACGG-3′ and 5′-AAACCGTCGTTGAGCAGTTTGGTGC-3′. Synthesized oligonucleotides for target KO gRNA were annealed and cloned at SapI sites into the pCGSapI vector, which was then transfected into JM109 cells. Clones were purified and subjected to DNA sequence analysis to check the oligonucleotide insert (Eurofins, Japan). Selection markers for cell transfection were neomycin (G418), hygromycin, and blasticidin resistance genes.

### 2.2 Cell transfection

MDCK cells were cultured in Dulbecco's modified Eagle's medium (DMEM) containing 10% of fetal calf serum. The cells were transfected with 10 µg of the expression vectors or KO vectors by the calcium phosphate method [19]. To construct the β-catenin KO MDCK cells (termed β-cat KO cells), MDCK cells were cotransfected with pCGSapI-β-catenin gRNA and the blasticidin resistance gene. After 48 h of transfection, the cells were subjected to selection with blasticidin S (8 µg/ml). Single colonies were isolated and analyzed by immunofluorescent staining and immunoblotting with an anti–β-catenin antibody. To prepare cells with a double KO (DKO) of β-catenin and γ-catenin (termed βγ-DKO cells), β-cat KO cells were cotransfected with pCGSapI-γ-catenin gRNA and the hygromycin resistance gene. After selection with hygromycin B (300 µg/ml), single colonies were isolated and analyzed by immunofluorescent staining and immunoblotting with an anti–γ-catenin antibody. To obtain LEF-1–overexpressing cells, β-cat KO and/or βγ-DKO cells were transfected with the LEF-1 expression vector. After selection with G418, single colonies were isolated and analyzed by immunofluorescent staining and immunoblotting with an anti-HA antibody. Only one β-cat KO clone was isolated, but others were at least two independent clones that yielded essentially the same results.

### 2.3 Antibodies

Mouse monoclonal antibodies (mAbs) against E-cadherin, β-catenin, γ-catenin, fibronectin, and p120-catenin were purchased from Transduction Laboratories (Lexington, KY). A mouse mAb against vinculin and a rat mAb against HA were bought from Sigma (St. Louis, MO) and Roche Diagnostics GmbH (Mannheim, Germany), respectively. A rabbit mAb against Slug was acquired from Cell Signaling Technology Japan (Tokyo, Japan). A goat polyclonal antibody against ZEB1 was purchased from Santa Cruz Biotechnology (Santa Cruz, CA). All secondary antibodies were acquired from Jackson ImmunoResearch Laboratories (West Grove, PA).

### 2.4 Immunofluorescence microscopy

Cells were grown on coverslips for 48 h, fixed with 3% paraformaldehyde in PBS for 20 min, and permeabilized with 0.1% Triton X-100 in PBS. After blocking, the fixed cells were incubated with primary and secondary antibodies as described previously [19] and analyzed under an Olympus fluorescence microscope (Tokyo, Japan) equipped with a CD72 camera (Olympus).

### 2.5 Immunoblotting

Immunoblot analysis was carried out as described elsewhere [19]. Briefly, cells were lysed, and proteins were separated by polyacrylamide gel electrophoresis and transferred to a nitrocellulose membrane. The membranes were blocked with 5% nonfat milk and then incubated with primary antibodies for either 2 h at room temperature or overnight at 4 °C, followed by incubation with a peroxidase-conjugated secondary antibody. Bound antibodies were visualized by enhanced chemiluminescence (ECL; Amersham International, Little Chalfont, UK).

### 2.6 Genomic-DNA extraction

Each KO cell line was lysed in TNE buffer containing 0.5% SDS, collected, and supplemented with 200 µg/ml proteinase K (Wako, Japan), with incubation at 50 °C for 3 h. Equal volumes of phenol and chloroform were added, and the mixture was rotated at 4 °C for 30 min; this procedure was performed twice. After centrifugation, supernatants were collected and mixed with a double volume of EtOH. The precipitate was dissolved in TE buffer containing 20 µg/ml DNase-free RNase (Sigma, St. Louis, MO), with incubation at 37 °C for 30 min. After the second precipitation with EtOH and centrifugation, the precipitates were dissolved in TE buffer and employed as a template for PCR.

### 2.7 PCRs of genomic DNA

These procedures were carried out and confirmed β-catenin and γ-catenin gene KOs. GoTaq DNA polymerase (Promega, Madison, WI) was used for PCRs. PCR conditions were optimized for each primer pair as previously described [20]. The primer pairs were as follows: β-catenin KO, forward, 5′-TGGTGGTTAATAAGGCTGCAG-3′ and reverse, 5′-TAAAGATGGCCAGCAAGCCC-3′; γ-catenin KO, forward, 5′-AGCTGCTCAAGTCAGCCATC-3′ and reverse, 5′-TCCACAAAGTTCAGAGGGAC-3′. PCR products were cloned into the *Sma*I site of the pBluescript II KS (+) vector. After transformation, plasmids were purified and subjected to genomic-DNA sequence analysis (Eurofins, Japan).

## 3 Results and discussion

### 3.1 Expression of LEF-1 induces EMT in β-catenin KO MDCK cells

In our previous study, we have reported that not only wild-type LEF-1 but also an amino terminus–deleted mutant LEF-1, ΔNLEF-1, which cannot interact with β-catenin, has an ability to induce EMT [12]. From this result, we concluded that β-catenin was not necessary for the induction of EMT. Nonetheless, in mesenchymal and osteoblastic cells, ΔNLEF-1 reportedly retains the ability to interact with β-catenin at the site corresponding to residues 150–175 [13].

**Fig. 1:**
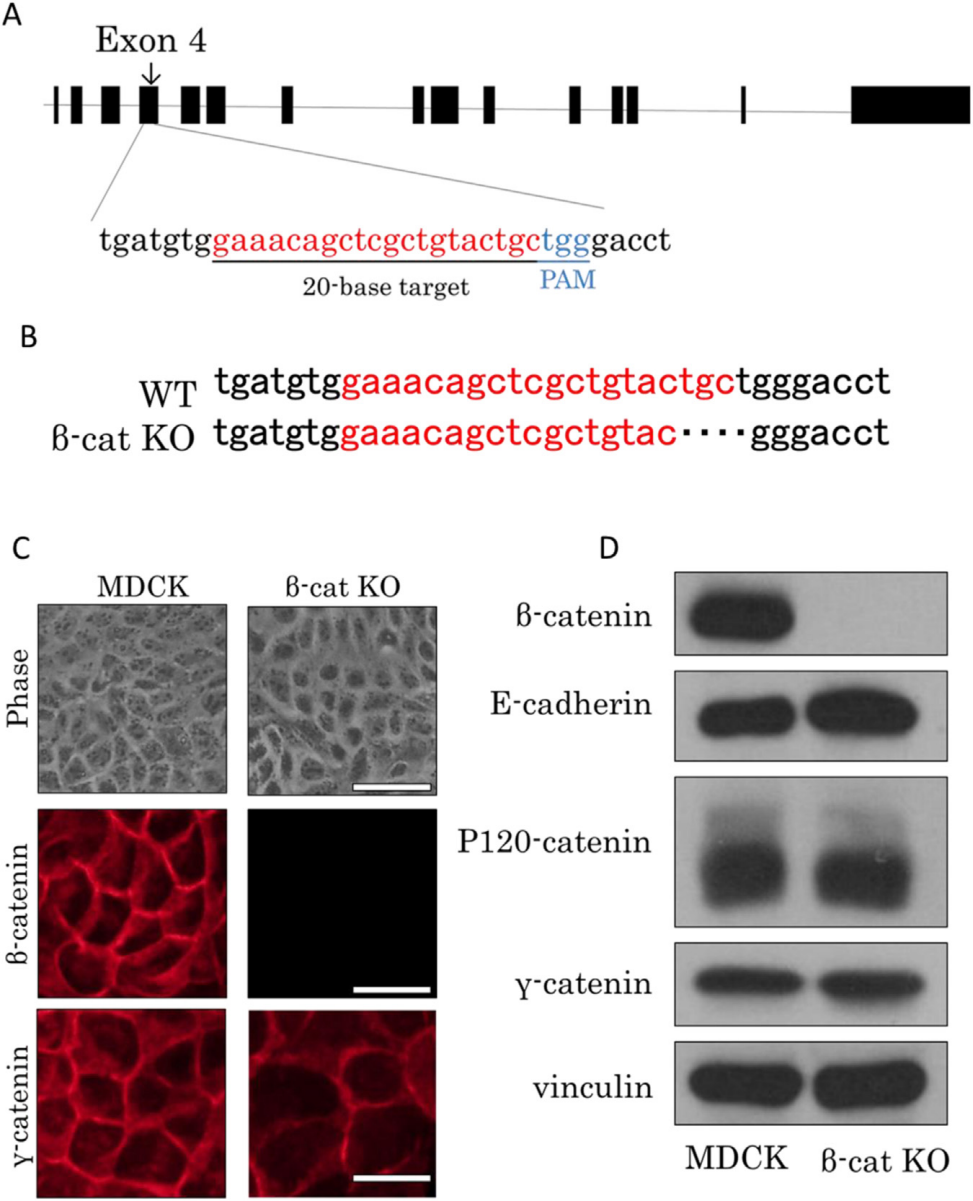
The knockout of the β-catenin gene in MDCK cells. (A) Schematic representation and a position of the candidate gRNA target site in exon 4 of the β-catenin gene (*CTNNB1*). Targeted and PAM sequences are red and blue, respectively. (B) Genome sequence analysis. Four-nucleotide sequences are deleted in β-catenin knockout cells. At least eight genome sequences of cell clones were analyzed, and all clones yielded the same results. (C) Cell morphology and immunofluorescent staining. Phase contrast microscopy shows that β-cat KO cells have no morphological changes as compared with parental MDCK cells. Immunofluorescent staining using the anti–β-catenin antibody showed that β-catenin was undetectable in β-cat KO cells, but γ-catenin was still present at the plasma membrane. Scale bar, 50 µm. (D) Immunoblot analysis of β-catenin, E-cadherin, p120-catenin, and γ-catenin in MDCK cells and β-cat KO cells. Vinculin served as a loading control.

To determine whether β-catenin is required for induction of LEF-1–induced EMT, we disrupted the β-catenin gene using the CRISPR/Cas9 gene editing system. We targeted the 20-nucleotide sequences upstream of the protospacer-adjacent motif (PAM) sequence in exon 4 of the canine β-catenin gene (canine *CTNNB1*; Fig. 1A). The candidate gRNA sequence was inserted at the SapI site into the pCGSapI vector [18], and pCGSapI-β-cat gRNA was subjected to sequence analysis to confirm the inserted oligonucleotide. The KO vector was cotransfected with the blasticidin resistance gene into MDCK cells to generate stable transfectants after selection with blasticidin S. We isolated a β-catenin–negative clone detected by immunofluorescent staining (Fig. 1C, middle panels) and immunoblot analysis (Fig. 1D) with the anti–β-catenin antibody. The negative clone then underwent extraction of genomic DNA, and we amplified the region including the gRNA target site. PCR products were cloned into the pBluescript II KS (+) vector and subjected to genomic DNA sequence analysis. The clones showed 4-base deletions adjacent to the PAM site (Fig. 1B), and thus we successfully obtained β-catenin KO MDCK cells (termed β-cat KO cells). Phase contrast microscopy indicated that the morphology of β-cat KO cells was indistinguishable from that of parental MDCK cells (Fig. 1C, upper panel). Immunoblot analysis showed the same expression level of E-cadherin, an epithelial marker, and the same expression of an alternative splicing variant: p120-catenin (Fig. 1D). The expression of γ-catenin, a close homolog of β-catenin, was localized to the juxtamembrane space in β-cat KO cells (Fig. 1C, lower panel).

**Fig. 2:**
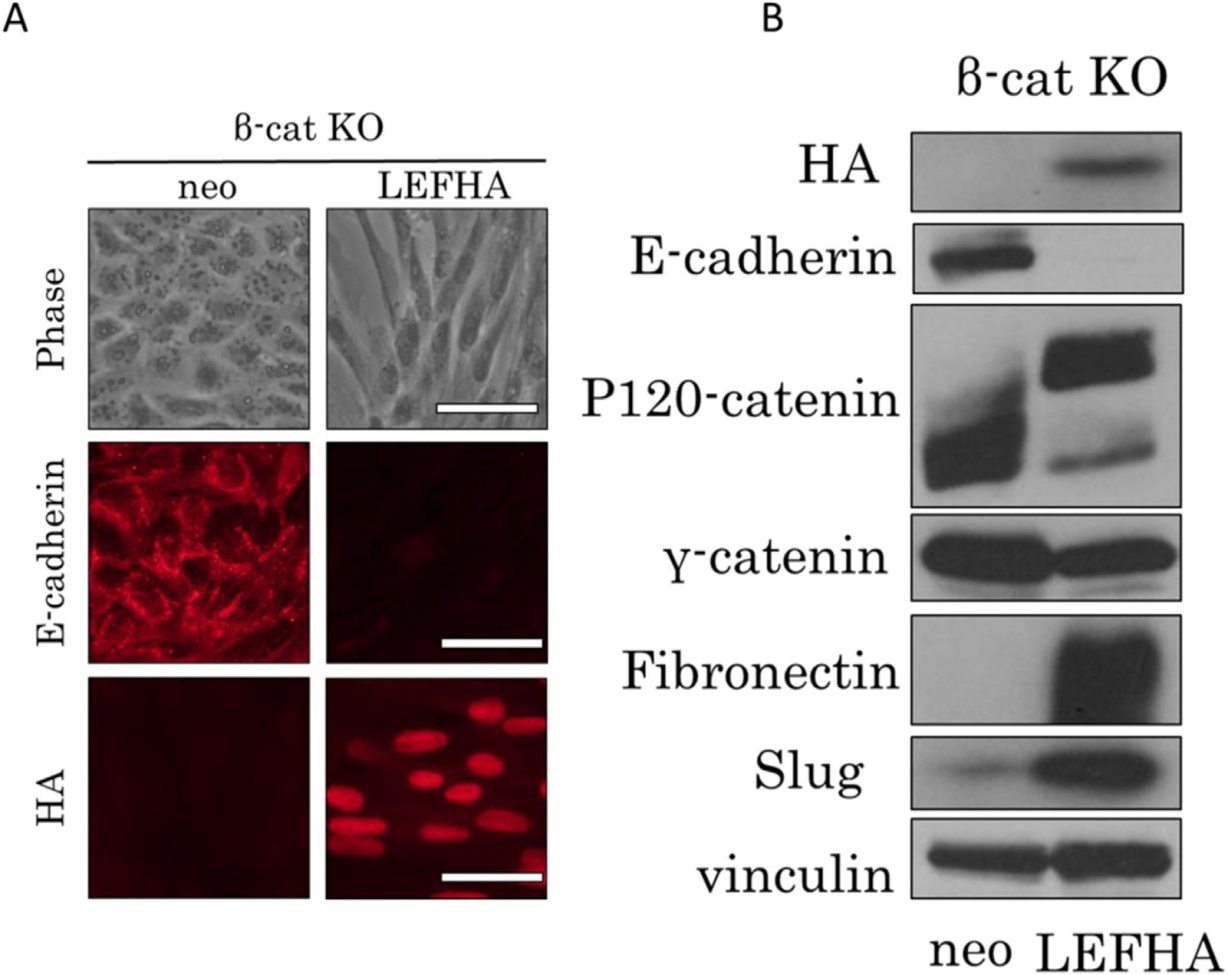
Overexpression of LEF-1 induces EMT in β-catenin KO cells. (A) Cell morphology and immunofluorescent staining. Phase contrast microscopy shows that the expression of LEF-1 induces morphological changes, from an epithelial to fibroblastic phenotype. Staining with the anti-HA antibody revealed that LEF-1 was localized in the nucleus. E-cadherin was not detected in LEF-1–expressing cells. “Neo” means that only the neomycin resistance gene was introduced and denotes control cells. (B) Immunoblot analysis revealed that the expression of LEF-1 in β-cat KO cells downregulated E-cadherin, upregulated fibronectin, and caused changes in the pattern of p120-catenin splicing reflective of EMT. Slug was also upregulated. Vinculin served as a loading control.

Next, we introduced a hemagglutinin (HA)-tagged mouse LEF-1 expression vector into β-cat KO cells to determine whether LEF-1 can induce EMT without β-catenin. As expected, expression of LEF-1 induced morphological changes in β-cat KO cells toward the fibroblastic phenotype (Fig. 2A). Immunoblot analysis revealed molecular changes including downregulated expression of E-cadherin, alteration of p120-catenin splicing, and upregulation of mesenchymal markers, fibronectin, and EMT-related transcription factor Slug (Fig. 2B). Taken together, these results supported our hypothesis that LEF-1 can induce EMT in MDCK cells independently of β-catenin.

### 3.2 LEF-1 does not require γ-catenin in β-cat KO cells to induce EMT

Some studies have shown that γ-catenin too has an ability to bind LEF-1 [21]. Moreover, γ-catenin can compensate the loss of β-catenin in Wnt signaling [16] and cell adhesion [17]. To examine the possible participation of γ-catenin in the induction of EMT by LEF-1 expression, we disrupted the γ-catenin gene by means of the CRISPR/Cas9 system.

**Fig. 3:**
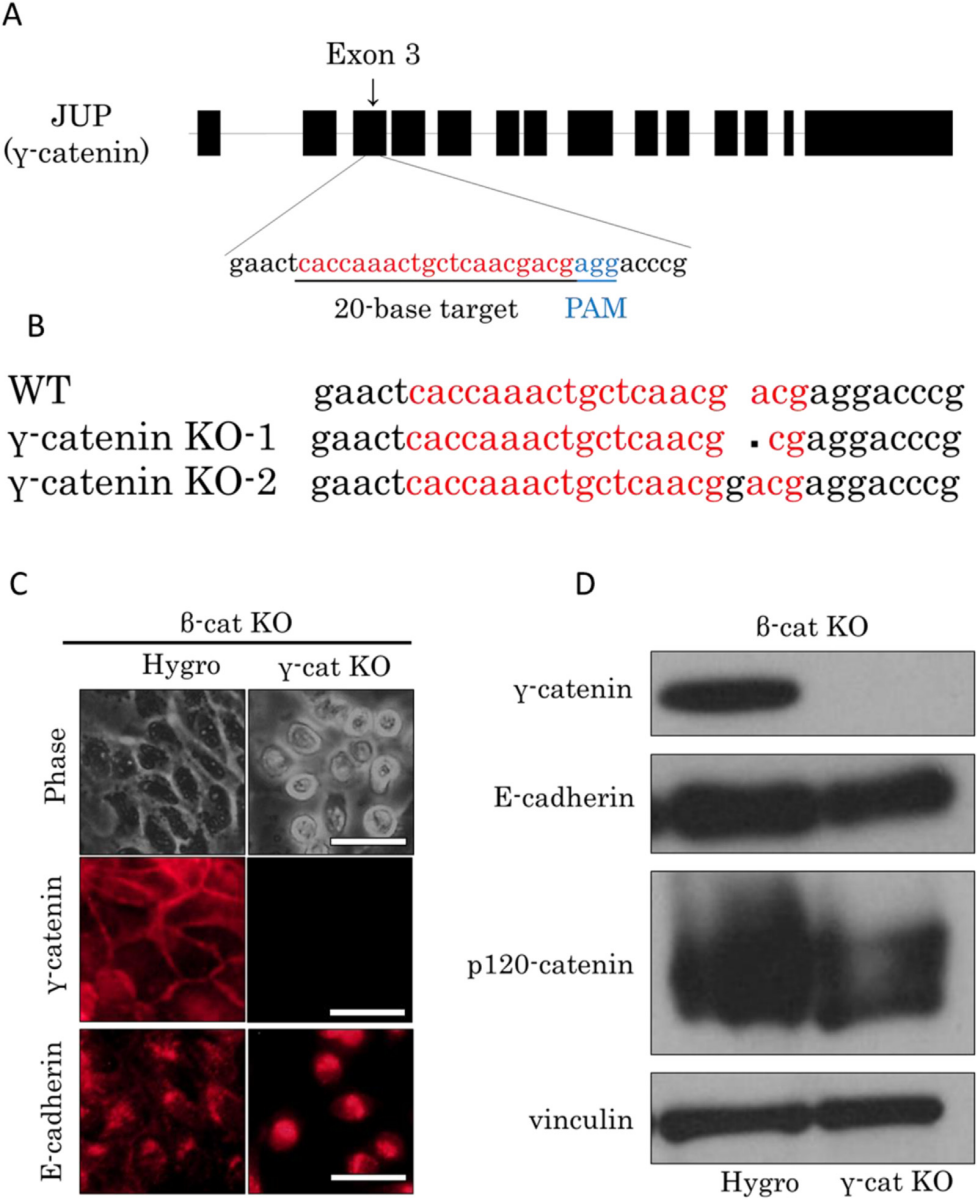
The β-catenin γ-catenin DKO cells kept epithelial molecules. (A) Schematic representation and position of the gRNA target site in exon 3 of the γ-catenin gene (*JUP*). Targeted and PAM sequences are red and blue, respectively. (B) Genome sequence analysis. Two γ-catenin–negative clones were isolated, and extracted genomic DNA was sequenced. One clone showed a 1-nucleotide deletion in the proximity of the PAM site, and another clone showed a 1-nucleotide insertion. At least eight genome sequences of cell clones were analyzed, and all the clones gave the same results. (C) Cell morphology and immunofluorescent staining. Phase contrast microscopy shows that βγ-DKO cells acquired spheroidal morphology. Immunofluorescent staining using the anti–γ-catenin or anti–E-cadherin antibody. “Hygro” means that only the hygromycin resistance gene was transfected and denotes control cells. Scale bar, 50 µm. (D) Immunoblot analysis revealed that the βγ-DKO cells lost γ-catenin expression but retained the expression of E-cadherin and showed no changes in the p120-catenin splicing pattern. Vinculin served as a loading control.

To perform the KO of γ-catenin, we searched for a target site (20-nucleotide sequence) upstream of the PAM sequence in exon 3 of the canine γ-catenin gene (canine *JUP*; Fig. 3A). The candidate synthetic gRNA gene was inserted at the SapI site into the pCGSapI vector, and pCGSapI-γ-cat gRNA was verified by sequence analysis to confirm the inserted oligonucleotide. The γ-catenin KO vector was cotransfected with the hygromycin resistance gene into the β-cat KO cells to generate stable transfectants after selection with hygromycin B. We isolated some γ-catenin–negative clones detected by immunofluorescent staining (Fig. 3C, middle panels) and immunoblot analysis (Fig. 3D) with the anti–γ-catenin antibody. The genomic DNA was extracted from γ-catenin–negative clones and processed for sequence analysis. One clone showed a 1-base deletion near the PAM site, and another clone showed a 1-base insertion (Fig. 3B). These mutations sequentially caused a frameshift, and both established clones turned out to be successful β-catenin γ-catenin DKO cells (termed βγ-DKO cells).

Although MDCK cells with a single β-catenin KO retained epithelial morphology (Fig. 1C and Fig. 3C), βγ-DKO cells showed reduced cell–cell contacts and manifested spheroidal morphology (Fig. 3C). The level of E-cadherin expression was retained, and p120-catenin splicing was not altered as compared with the control cells (Fig. 3D). These observations concerning cell morphology are consistent with previous experiments on F9 embryonal carcinoma cells [17]. The role of β-catenin as an adaptor between E-cadherin and α-catenin can be replaced by γ-catenin. Therefore, only βγ-DKO cells show morphological changes. Consistent with these observations, α-catenin KO MDCK cells showed disrupted cell-cell adhesion but the amounts of E-cadherins was not altered [22].

**Fig. 4:**
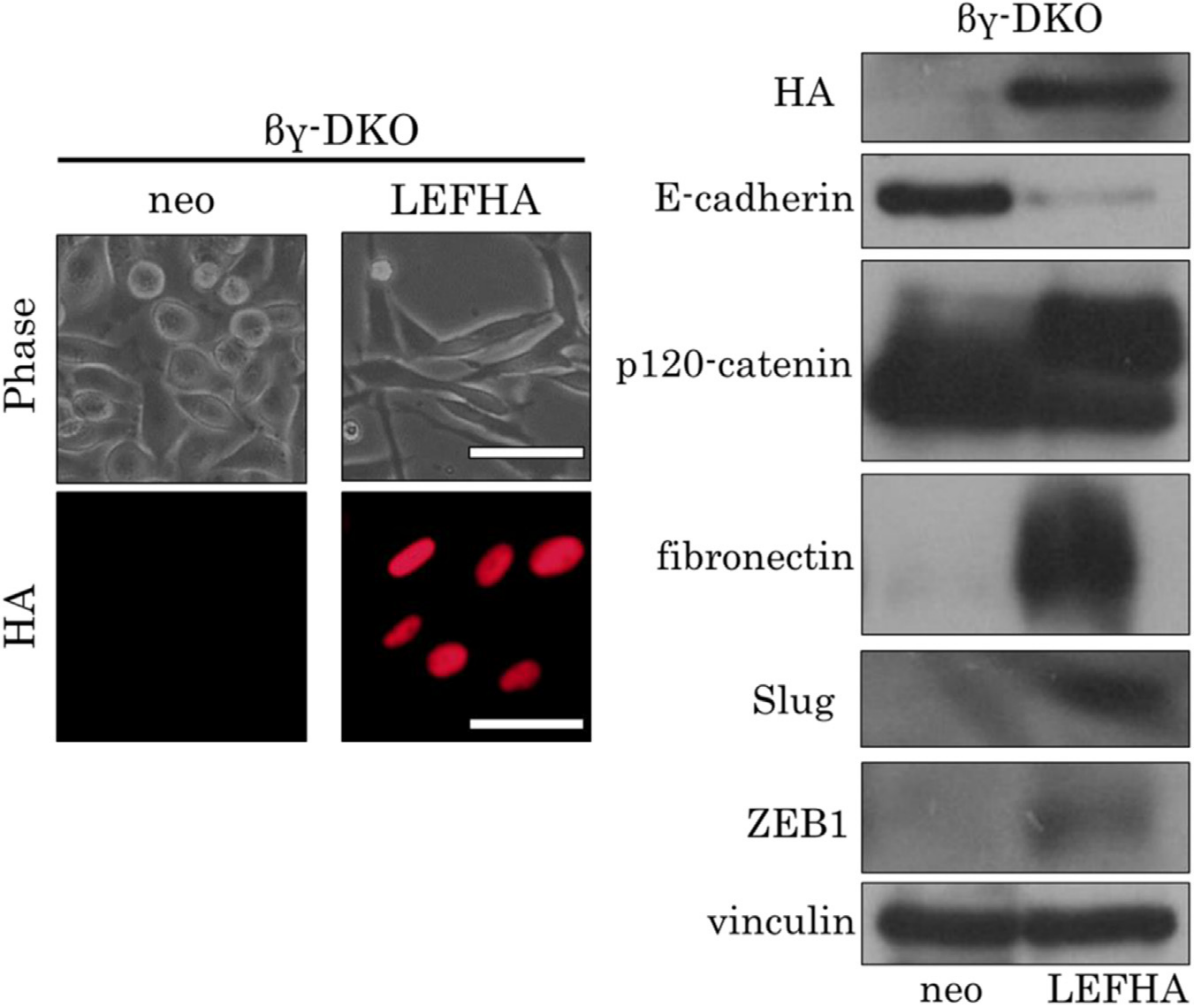
Expression of γ-catenin is not essential for induction of EMT by LEF-1 expression. (A) Phase contrast microscopy shows that overexpression of LEF-1 in βγ-DKO cells induced morphological changes: from a spheroid to fibroblastic phenotype. Immunofluorescent staining with the anti-HA antibody showed nuclear localization of LEF-1. “Neo” means that only the neomycin resistance gene was introduced and denotes control cells. Scale bar, 50 µm. (B) Immunoblot analysis revealed that expression of LEF-1 downregulated E-cadherin and caused changes in the splicing pattern of p120-catenin reflective of EMT, and upregulated fibronectin, Slug, and ZEB1. Vinculin served as a loading control.

Next, we tested whether LEF-1 induces EMT in these cells: the LEF-1 expression vector was introduced in βγ-DKO cells, and we isolated HA-positive clones with the anti-HA antibody (Fig. 4A). The cells expressing LEF-1 (LEF/βγ-DKO cells) showed morphological changes, including transition from epithelial to spheroidal or fibroblastic morphology (Fig. 4A, upper panel). Immunoblot analysis revealed that in βγ-DKO cells, the expression of E-cadherin was downregulated, p120-catenin splicing was altered, and fibronectin, Slug, and ZEB1 were upregulated (Fig. 4B). These results indicated that LEF-1 expression in βγ-DKO cells can induce EMT. Thus, γ-catenin was not required for induction of EMT either. Consequently, in MDCK cells, β-catenin and γ-catenin were unnecessary for LEF-1 to induce EMT.

Despite the finding that transcription factor LEF-1 interacts with β-catenin to activate their target genes, such as EMT-related gene *SNAI2* [7], and induces EMT, we found that not only full-length LEF-1 but also LEF-1 lacking the N-terminal β-catenin–binding domain can induce EMT. These data suggested that LEF-1 may induce EMT independently of β-catenin [12]. LEF-1 with the amino-terminal deletion, however, seems to retain the ability to interact with β-catenin via another region: residues 150–175 [13]. Therefore, the question remains whether β-catenin is required for LEF-1-induced EMT. To answer the question, we created MDCK cells with a knockout of the β-catenin gene. We obtained evidence that LEF-1 can induce EMT in the absence of β-catenin. Furthermore, using β-catenin γ-catenin DKO cells, we showed that not only β-catenin but also γ-catenin is unnecessary for the induction of EMT.

There are some reports that EMT-related transcription factor Slug and ZEB1 are encoded by some of the target genes of Wnt/β-catenin signaling [7], [23]. Consistent with our previous study, in the present research, the level of Slug or ZEB1 expression was upregulated in β-cat KO and βγ-DKO cells. These results suggest that Slug and ZEB1 are target genes of LEF-1, but their expression takes place in the absence of β-catenin in MDCK cells. A molecule(s) that interacts with LEF-1 and is involved in induction of EMT by LEF-1 will be identified in the next study.

In conclusion, we provided clear evidence that LEF-1 induces EMT in a β-catenin–independent manner. Identification of the molecule(s) interacting with LEF-1 via a site other than the N-terminal region is necessary for understanding the mechanism of EMT induction by LEF-1.
